# Malpositioned Suprapubic Catheter Results in Perforation of Penile Urethra and Septic Shock

**DOI:** 10.7759/cureus.6965

**Published:** 2020-02-12

**Authors:** Steven Ferrell, R. Erik Connor

**Affiliations:** 1 Emergency Medicine, Brooke Army Medical Center, San Antonio, USA

**Keywords:** suprapubic catheter, penile perforation, sepsis

## Abstract

Placement of suprapubic catheters (SPCs) has a relatively low complication rate and is well tolerated by most patients. Most complications take place during initial catheter placement or during replacement. Malposition of the catheter may cause serious damage to the bowel, bladder, or urethra. Care must be taken to ensure proper placement and functionality by the provider. We describe the case of a 66-year-old male with a history of neurogenic bladder and indwelling SPC presenting to the emergency department 36 hours after catheter replacement with bright red blood from the penile urethra, abdominal pain, fever, and hypotension. Computed tomography scan revealed that during replacement the SPC had passed into the penile urethra, with the bulb fully inflated causing rupture of the urethra with resultant sepsis. This case illustrates the importance of confirming SPC placement prior to bulb inflation and patient discharge.

## Introduction

Suprapubic catheters (SPCs) are a safe and effective alternative to long-term urethral catheterization and are routinely placed for patients requiring extended catheter placement [1,2]. SPCs require frequent replacement, which is often completed in the office setting by physicians, nurses, or technicians. Complications from long-term use are most commonly related to urinary infections (21%) and less frequently bowel injury due to catheter malposition (2.7%) [2]. Care must be taken to ensure proper placement of the catheter prior to bulb inflation and patient discharge to prevent damage to the bladder, urethra, or bowel. We present the case of a 66-year-old male with an SPC presenting to the emergency department (ED) with abdominal pain, bleeding from the penile urethra, bladder distention, fever, and hypotension status post SPC replacement 36 hours prior. The patient’s symptoms were caused by an SPC that was malpositioned in the base of the penile urethra, causing perforation of the urethra with resulting sepsis. 

## Case presentation

A 66-year-old African American male with a history of neurogenic bladder requiring SPC presented to the ED complaining of fever, fatigue, abdominal pain, bladder distention, and bleeding from his penis. The patient reported that his SPC was replaced at his urologists’ office 36 hours prior and his symptoms started shortly thereafter. The patient admits to having no urinary output from his catheter for the past 36 hours and was concerned the line was clogged. He noted that he does not typically have penile discharge but has had profuse bright red bleeding from his penis that began shortly after his catheter was replaced and has been increasing in intensity. The patient’s comorbidities include C3-4 fracture (traumatic), neurogenic bladder, incomplete paraplegia, and hypertension.

Vital signs on presentation to the ED included a blood pressure (BP) 146/90 millimeters of mercury (mmHg), a heart rate of 137 beats/minute, a respiratory rate of 19 breaths per minute, an oxygen saturation of 95% on room air, and a temperature of 38.0 degrees Celsius. Upon initial evaluation by the emergency physician (EP), the patient's temperature had increased to 39.1 degrees Celsius and he was in severe pain. Physical exam revealed severe abdominal tenderness diffusely with guarding and notable distention. A moderate amount of bright-red urethral discharge was noted while the remaining genital exam was unremarkable. A pre-existing bilateral lower extremity sensory deficit was present and related to a prior spinal cord injury. The remainder of the physical exam was unremarkable.

Laboratory studies were notable for a white blood cell count of 13.6, lactate of 2.4 mmol/L, urinalysis with large blood, and trace leukocyte esterase. The complete metabolic panel and venous blood gas were both within normal limits. Urine and blood cultures were obtained. 

The patient was treated with an initial 500 ml bolus of intravenous (IV) PlasmaLyte and 50 micrograms of Fentanyl for the abdominal pain. With an immediate concern for sepsis the patient was given 3.375 grams (gm) of Zosyn, 1 gm of vancomycin, in addition to 1 gm of IV Tylenol for the fever.

**Figure 1:**
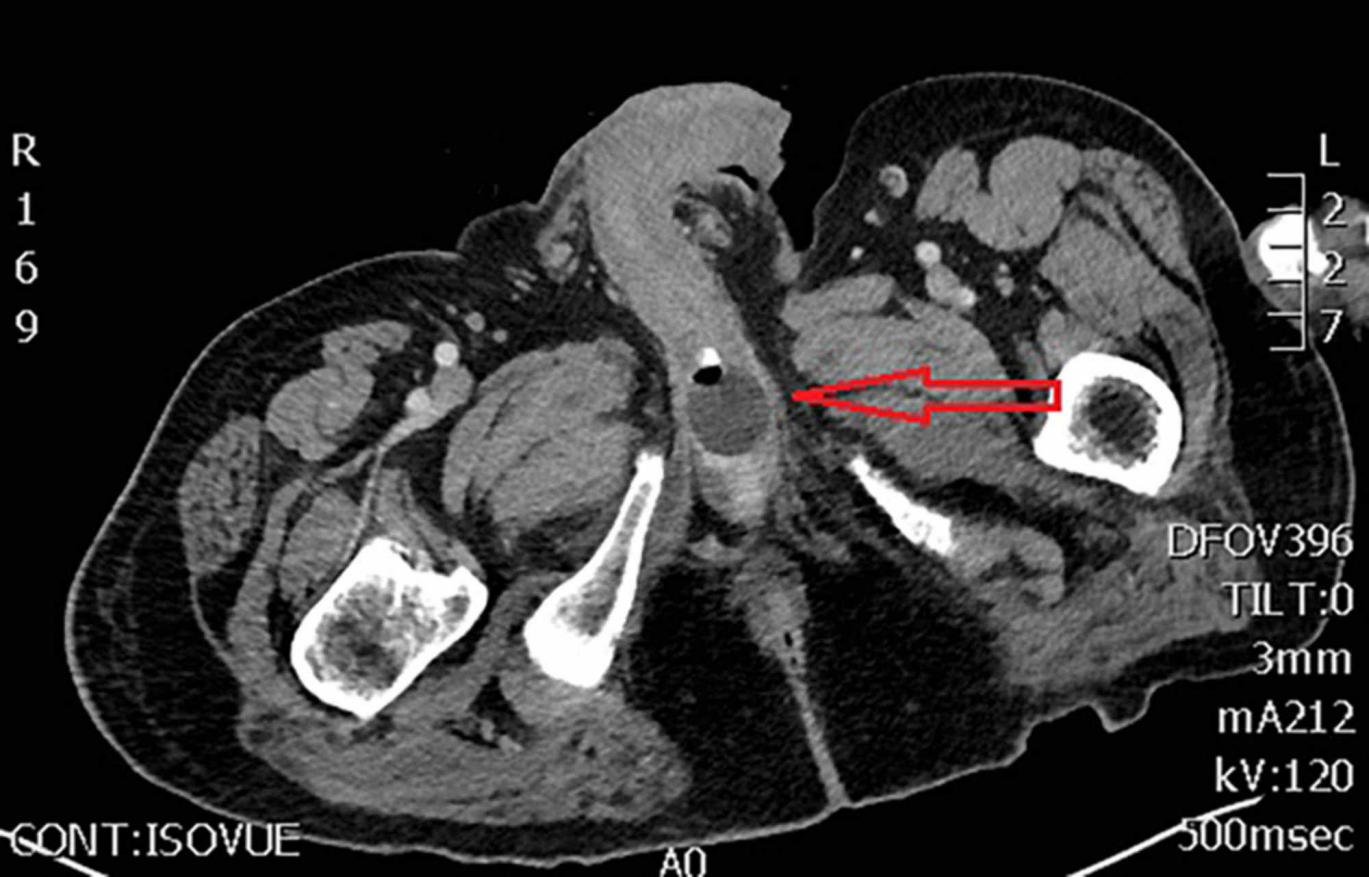
Axial CT scan with the arrow indicating inflated catheter in the penile urethra.

**Figure 2:**
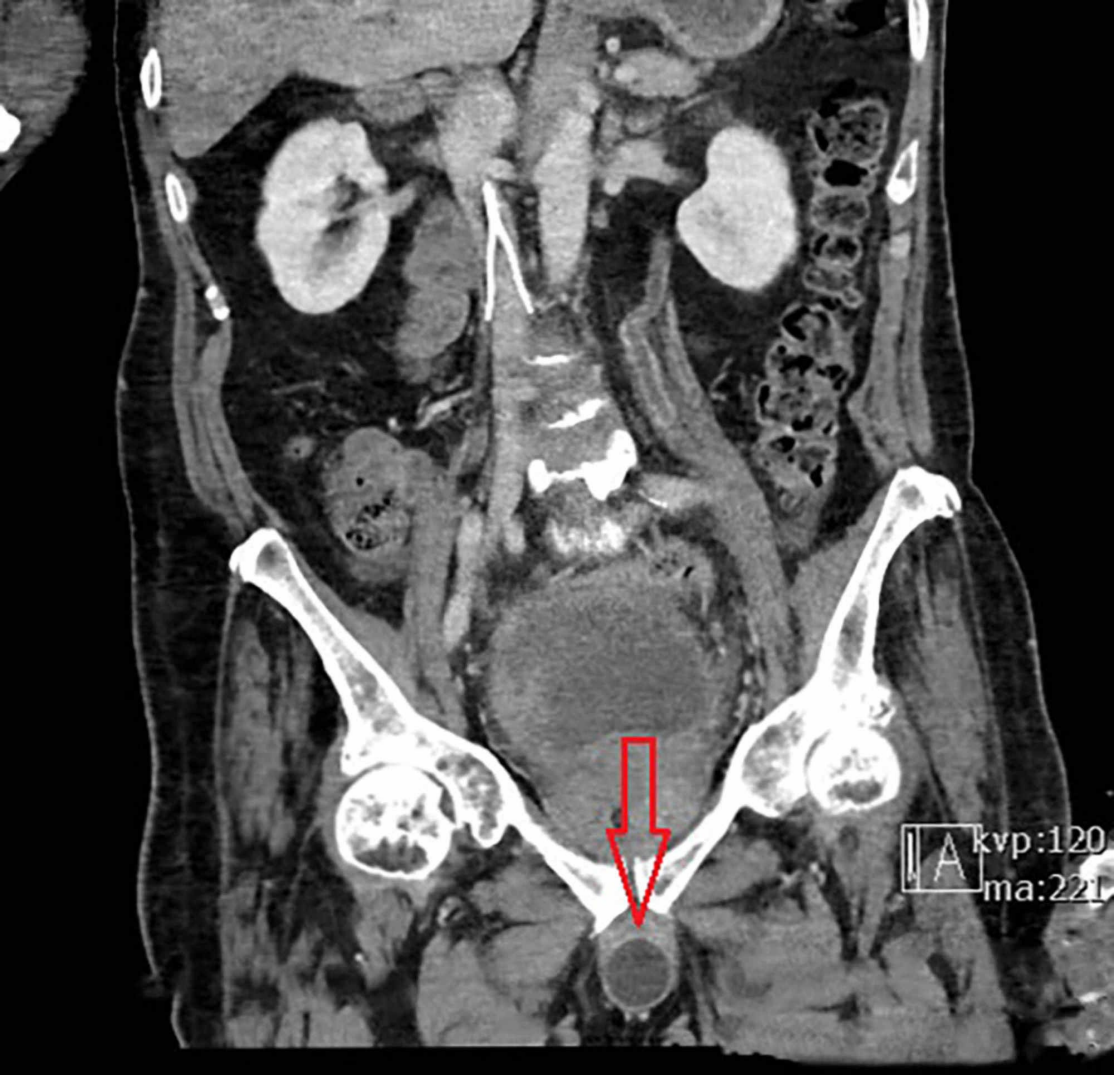
Coronal CT scan with the arrow indicating inflated catheter in penile urethra.

A computed tomography (CT) scan of the abdomen/pelvis with IV contrast was ordered to further evaluate the cause of the abdominal pain and distention. The EP received a call from the radiologist noting that the SPC balloon was inflated and positioned in the penile urethra. The final CT read was “Malpositioned suprapubic catheter with balloon tip in the penile urethra. Some surrounding hyperdense fluid, likely hemorrhage around the balloon tip. Catheter tip appears likely perforating the penile urethra" (Figures 1, 2). Urology was immediately consulted and the EP was instructed to deflate the balloon and pull the catheter back until urine flow was present in the tubing. The balloon was deflated and pulled back, which resulted in 600 cc of red urine output and provided the patient with moderate pain relief. The urologist evaluated the patient and concluded that during catheter replacement the previous day it inadvertently inserted into penile urethra, where the balloon was inflated, resulting in perforation.

While the patient’s pain began to resolve after the catheter was repositioned and his bladder drained, his BP decreased to a mean arterial pressure of 60 mmHg despite a two liter bolus of PlasmaLyte. The patient was admitted to the medical intensive care unit where he required 12 hours of norepinephrine vasopressor support before his BP stabilized. One day after admission, he transferred to a step-down unit where he remained for seven days of continued antibiotic treatment prior to discharge. The patient’s urine culture resulted in growth of Staphylococcus aureus, Proteus mirabilis, and Enterococcus faecalis for which he received seven days of IV Zosyn during his hospital course. The patient had no prior history of urine colonization of Staphylococcus aureus or Proteus mirabilis. He was discharged with urology follow-up.

## Discussion

SPCs are indicated in patients who are unable to tolerate urethral catheters, have existing prostate obstruction, or in patients undergoing pelvic or urethral surgery [1]. SPC placement is a good alternative to standard urethral catheterization for patients with neurogenic bladder dysfunction as seen in patients with a history of stroke, spinal trauma, and Parkinson’s disease [1,2]. While SPCs are typically placed by a urologist, they may also be placed emergently in cases of trauma to the pelvis or urethra by a surgeon or EP when urethral catheterization is contraindicated. SPCs are placed using the Seldinger technique where the catheter is fed over a wire through the abdominal wall into the bladder often using ultrasound for guidance [3]. Catheters range in size from 10 to 18 F. First time non-emergent SPCs are placed under general or local anesthesia in an operating room while catheter replacements are often done in an office setting or at home. Catheters are typically replaced every 4-10 weeks [1]. SPCs are preferred for patients who need long-term catheterization as they are better tolerated when compared to traditional urethral catheters [2,4].

While most patients typically tolerate long-term SPCs, complications can occur both during initial surgical placement and from resultant catheter changes [5]. Common complications include infection, bleeding, bladder injury, small bowel obstruction, bowel perforation, and in rare cases urethral injury due to misplacement as was observed in this case [2]. Initial catheter placement has a reported complication rate of 10% with the most serious complication of bowel injury occurring in 2.7% of the cases [2,6]. Patients with neurogenic bladder or significant comorbidities have the highest risk of complications. Patients with long-term SPC placement are prone to urinary infections and often become permanently colonized; 21% of the patients with an SPC suffer from recurrent urinary tract infections [2]. Urinary infection is the most common reason patients with an SPC seek care in the ED. Patients should be treated with antimicrobial therapy based on prior urinary cultures, in conjunction with the patient's urologist [6]. Urinary infections resulting in sepsis have been reported at a rate as high as 9% from long-term SPC use [2]. Small bowel obstruction may develop soon after SPC placement or may have a delayed presentation occurring several weeks status after surgery. Bowel and bladder perforation is rare and most often happens during surgical placement but has been reported in patients during catheter replacement [2,3]. Malposition of the catheter into the urethra with resultant perforation and sepsis as in our case is extremely rare, and no similar cases were identified during a literature search.

Catheter replacement is a routine procedure with a low complication rate when done correctly. Verifying proper catheter placement is imperative prior to discharging a patient. As in our case, many patients with neurogenic bladder or paralysis may not feel associated pain of a malpositioned device but will present with other associated symptoms such as bleeding, fever, hypotension from sepsis, bladder distention, or referred pain [6]. This novel complication may result in significant morbidity or mortality and is imperative to recognize and treat immediately.

## Conclusions

SPCs are a safe alternative to urethral catherization and are better tolerated in patients needing long-term catheter placement. Serious complications are uncommon but may result in serious illness or death in rare cases. As these patients most often will present to the ED, it is imperative for the EP to have a basic understanding of SPC placement, removal, and associated complications. As seen in the presented case, confirming proper catheter balloon placement is of the highest importance prior to patient disposition. 
